# Assessing patterns, barriers, and motivations for family planning utilization among currently pregnant women in Nigeria: a cross-sectional study

**DOI:** 10.3389/frph.2026.1789800

**Published:** 2026-05-21

**Authors:** Ayodamola Bakare, Kofoworola Akinsola, Julius Salako, Olabisi Olasupo, Carina King, Adegoke G. Falade, Ayobami A. Bakare

**Affiliations:** 1 Department of Paediatrics, University of Ibadan, Ibadan, Oyo, Nigeria; 2 Institute for Global Health, University College London, London, United Kingdom; 3 Department of Global Public Health, Karolinska Institutet, Stockholm, Sweden; 4 Department of Paediatrics, University College Hospital, Ibadan, Oyo, Nigeria; 5 Department of Community Medicine, University College Hospital, Ibadan, Oyo, Nigeria

**Keywords:** gender equality, maternal health literacy, postpartum contraception, reproductive health, women's empowerment

## Abstract

**Introduction:**

Universal access to family planning is critical to reducing maternal mortality, yet progress remains slow, particularly in Sub-Saharan Africa. In Nigeria, modern contraceptive use is 12%, compared to a global average of 77.5%. This study, therefore assessed contraceptive use patterns, barriers, and motivations among pregnant women to inform the design of context-specific interventions aimed at improving family planning uptake.

**Methods:**

We conducted a cross-sectional study from July to September 2023 in different settings (Jigawa, Oyo, and Lagos States) to capture varying sociocultural contexts in Nigeria. Pregnant women attending antenatal care at 32 healthcare facilities were sampled using a multi-stage approach. Data collection was through structured questionnaires focusing on respondents' health literacy, family planning awareness and utilization. We used logistic regression to assess the relationship between family planning utilization and maternal health literacy and other sociodemographic characteristics, with statistical significance set at the 95% confidence level.

**Results:**

Overall, 52.7% of women reported ever using any family planning method after picture prompts, with notable regional differences: Lagos (66.4%), Oyo (61.0%) and Jigawa (35.0%). About one-third (31.9%) of pregnancies were unintended. The mean maternal health literacy (MHL) score was 34.4 (SD = 7.3), with Jigawa having the lowest mean score (30.1, SD = 6.4) compared to Lagos (36.8, SD = 6.3) and Oyo (37.5, SD = 6.9). Higher MHL scores (aOR = 1.32, 95% CI: 0.76–2.31), increasing number of living children (2–4 children: aOR = 2.93, 95% CI: 1.53–5.62), and residence in Lagos (aOR = 2.98, 95% CI: 1.27–7.01) were significantly associated with increased odds of family planning use.

**Conclusion:**

High maternal health literacy is associated with increased contraceptive utilization, but substantial obstacles, including social disapproval in Jigawa and accessibility challenges in Lagos, continue to impede family planning and adoption. These regional variations in contraceptive use call for targeted interventions to address specific local barriers and improve overall family planning outcomes.

## Introduction

Despite a 34% decline in global maternal mortality since 2,000, progress has stagnated, particularly in Sub-Saharan Africa. In 2020, 800 women died daily from pregnancy and childbirth complications (1–3). This is a maternal mortality ratio (MMR) of 545 deaths per 100,000 live births, compared to high-income countries where the MMR is as low as 4/100,000 live births (4). This inequity underscores the urgent need for expanded family planning access in Sub-Saharan Africa, where women face a 268 times higher lifetime risk of maternal death compared to Western Europe (5).

Sub-Saharan Africa has low access to and uptake of modern contraceptives (6), with only 24% of women ever using them (7). Enhancing family planning services could substantially lower maternal mortality rates, which remain a critical public health issue. Nigeria exemplifies this challenge. Progress in Nigeria has been gradual, with contraceptive use increasing only from 8% in 2000 to 20% in 2024, despite projections suggesting a potential rise to 35% by 2030 (8, 9). Currently, Nigeria's population growth rate stands at 2.4% (10) and its fertility rate is 5.2 children per woman (11–13), both figures surpassing global averages (14). Consequently, Nigeria is projected to become the world's third most populous country by 2050 (15). This is accompanied by a high maternal mortality ratio of 1,047 deaths per 100,000 live births in 2020, underscoring the urgent need for improved access to and utilization of family planning services (16–18), and sexual and reproductive health services more broadly (19).

Efforts to bolster family planning include initiatives like the Nigerian Urban Reproductive Health Initiative (NURHI), which has enhanced contraceptive supply within healthcare systems and boosted demand through community engagement and education. NURHI's success produced a 10% increase in modern contraceptive use and a rise in family planning interest from 2010 to 2014 (20), inspiring the creation of The Challenge Initiative (TCI) (21). TCI builds on NURHI's model by promoting state-level sustainability, encouraging local investments in financial and human resources for family planning services (22). Furthermore, Nigeria's revised National Policy on Population for Sustainable Development underscores a national commitment to expanding access to contraception and counseling. This initiative aims to stabilize population growth and enhance maternal health outcomes (23). Despite these ongoing efforts, only 12% of married women use modern contraceptive methods (13, 24, 25), a rate considerably lower than the global average of 77.5% (26, 27). Research underscores factors such as polygamy, early marriage, and significant age gaps in marriages as barriers to higher contraceptive use (24, 28, 29). Meanwhile, education has been highlighted as a crucial element in promoting family planning (25, 30).

Nevertheless, gaps persist in understanding the effect of maternal health literacy (MHL) on family planning utilization, limiting insights into usage patterns beyond initial determinants. National demographic surveys also indicate that access to contraception is low, and not sustained when it is used, as 41% of contraceptive users discontinue use within a year (13). Therefore, we assessed patterns, access barriers, and motivations for family planning use and non-use among currently pregnant women attending antenatal care clinics in three States in Nigeria. Our focus on currently pregnant women allows exploration of reluctance to use family planning methods prior to the current pregnancy, provides insights into regional barriers to family planning uptake, and reduces recall and social desirability biases. Moreover, pregnant women represent an important population for family planning research because antenatal care provides a critical opportunity for counselling on postpartum contraception and birth spacing.

## Methods

### Study design

We conducted a cross-sectional study, from July 1 to September 30, 2023, to investigate patterns, barriers, and motivation associated with prior family planning utilization/non-utilization among currently pregnant women in three States in Nigeria. We purposefully selected Jigawa, Oyo, and Lagos to capture sub-national variation in family planning practices. Jigawa, in the Northwest zone, represents an area with exceptionally low contraceptive use among women of reproductive age (only 4.0%). Conversely, the Southwest zone exhibits a significantly higher met need for family planning, with 49.4% and 22.6% in Lagos and Oyo, respectively.

### Study settings

Jigawa State is predominantly inhabited by the Hausa and Fulani peoples, who are mostly Muslim. Lagos and Oyo are predominantly occupied by the Yoruba people, with Christianity and Islam being the predominant religions. Lagos State, has a population exceeding 12 million, and the highest population density in Nigeria (31), while Oyo and Jigawa states have populations of 7.5 million and 6.7 million, respectively (32).

For our study, we purposefully selected 1–3 local government areas (LGAs) in each state: Kiyawa (rural) and Dutse (urban) in Jigawa State; Lagelu (peri-urban), Egbeda (peri-urban), and Ibadan southwest LGA (urban) in Oyo State; and Ikorodu LGA (peri-urban) in Lagos State. These areas represent diverse demographics and socioeconomic backgrounds. Kiyawa LGA in Jigawa has an estimated population of 230,000, with Dutse LGA housing around 202,448 inhabitants, primarily comprising Hausa and Fulani ethnic groups. They engaged in various occupations, including farming, trading, fishing, and civil service (33, 34). Lagelu LGA in Oyo consists of over 1,076 towns and villages, primarily engaged in farming activities such as palm oil and black soap production. Lagos State, predominantly inhabited by the Yoruba ethnic group, has the second-highest population in Nigeria (32). Ikorodu LGA, where the study was conducted, is a peri-urban setting, with an estimated population of over 1 million and an annual growth rate of 5.3% (35, 36).

### Study population and sampling technique

This study focused on pregnant women who utilized antenatal care (ANC) services at healthcare facilities. We focused on currently pregnant women to reduce recall and social desirability biases and gain further insight into patterns and practices around of family planning utilization. To achieve a representative sample across different regions, a multi-stage sampling approach was employed. First, a complete list of primary healthcare facilities (PHCs) serving the chosen LGAs was obtained from the State Ministry of Health. Any facilities lacking both antenatal care and delivery services were excluded from the list, and 32 facilities were then proportionally selected across five LGAs (12 each in Jigawa and Oyo states, and 8 from Lagos' Ikorodu LGA). The catchment areas served by these facilities functioned as clusters for the study.

Convenience sampling was employed for this study, resulting in a higher number of eligible participants from busier health facilities. The study included pregnant women aged 15 years and older who were not in critical condition during their visit and were proficient in English, Pidgin English, or one of the predominant local languages (Hausa/Fulani or Yoruba).

### Sample size

The sample size for the study was calculated using the formula for estimating a single proportion for an infinite population with a 95% confidence interval. In order to estimate 73.1% awareness of any family planning method (37), with a 5% margin of error requires a sample of 372. We therefore aimed to recruit 409 women to allow for 10% missing data and non-response in each state (38).

### Data collection

We recruited research assistants with at least a secondary education and fluency in both English and the local language predominant in the state (Yoruba/Hausa). After a 3-day training program, we conducted a 2-week pilot test at non-study facilities. Using Android tablets and Open Data Kit (ODK) software, the research assistants administered a structured questionnaire with sections on: demographics, awareness of family planning, ever use, immediate use before current pregnancy, intention to use after delivery and MHL. MHL used a 14-item maternal health literacy scale previously validated in Nigeria (39). We used picture prompts for the different family planning methods to help improve recall, including beads to demonstrate periodic abstinence, and notes arranged in a rectangular box to illustrate the withdrawal method. Data collection was done in 1–3 visits per facility cluster. Bi-monthly team meetings addressed any issues and ensured data cleaning and verification.

### Data management and analysis

We used summary statistics to describe the sociodemographic characteristics of the respondents. The primary exposure of interest in this study was MHL, with each of the 14-items scored on a 3-point Likert scale (Agree = 3, Neutral = 2, and Disagree = 1), with reverse scoring applied to four negatively worded items. A higher total score on this scale indicates a higher level of MHL. The primary outcome evaluated was the level of awareness and utilization of family planning methods. Awareness was defined as a binary variable, derived from the question “Are you aware of methods to delay or space childbirth or prevent unwanted pregnancy”. We assessed utilization of family planning methods using the question “Have you or your partner ever used any methods to delay or space childbirth”. A negative response to the question was taken as non-use of family planning methods Furthermore, we identified a list of traditional and modern family planning methods (40), and examined the proportion of pregnant women with family planning awareness, utilization patterns, and preferred methods. Multivariate logistic regression analysis was used to examine the relationship between ever use of family planning methods after picture prompt and women's sociodemographic characteristics. In the adjusted model, we controlled for maternal health literacy (MHL) scores and other relevant sociodemographic variables. Because religion and ethnicity were skewed across states, we included the state in the adjusted model and excluded religion and ethnicity to reduce potential multicollinearity. In addition, state is a more policy-relevant variable as state-based interventions are more feasible to implement in Nigeria compared to interventions along ethnicity or religion.

## Results

### Socio-demographic characteristics of participants

We recruited 548 participants from three Nigerian states for this study—Table 1. In Jigawa, 116 (53.5%) of the participants were aged 25–34, compared to 131 (63.0%) in Lagos and 74 (60.2%) in Oyo. Most participants were married across all states, but religion differed noticeably, with only 3.2% (7/217) being Christians in Jigawa vs. 62.5% in Lagos and 55.3% in Oyo. The highest level of education of women differs greatly between north and south, with 196 (94.3%) of participants in Lagos and 115 (93.5%) in Oyo having at least secondary education, compared to just 59 (27.2%) women in Jigawa (Table 1).

**Table 1:** Demographic characteristics of respondents (*N* = 548).

Variables	Jigawa (*n* = 217)	Lagos (*n* = 208)	Oyo (*n* = 123)	Total (*N* = 548)
	F (%)	F (%)	F (%)	F (%)
Age				
15–24	60 (27.6)	37 (17.8)	18 (14.6)	115 (21.0)
25–34	116 (53.5)	131 (63.0)	74 (60.2)	321 (58.6)
35 and above	41 (18.9)	40 (19.2)	31 (25.2)	112 (20.4)
Marital status				
Never married	0 (0.0)	11 (5.3)	2 (1.6)	13 (2.4)
Ever married	217 (100.0)	197 (94.7)	121 (98.4)	535 (97.6)
Religion				
Christianity	7 (3.2)	130 (62.5)	68 (55.3)	205 (37.4)
Islam	210 (96.8)	78 (37.5)	55 (44.7)	343 (62.6)
Ethnicity				
Hausa/Fulani	205 (94.5)	2 (1.0)	2 (1.6)	209 (38.1)
Igbo	3 (1.4)	30 (14.4)	5 (4.1)	38 (6.9)
Yoruba	3 (1.4)	162 (77.9)	110 (89.4)	275 (50.2)
Others	6 (2.7)	14 (6.7)	6 (4.9)	26 (4.7)
Woman Occupation				
Housewife/unemployed	152 (70.0)	36 (17.3)	8 (6.5)	196 (35.8)
Self employed	52 (24.0)	134 (64.4)	92 (74.8)	278 (50.7)
Employed	13 (6.0)	38 (18.3)	23 (18.7)	74 (13.5)
Husband's occupation				
Self employed	143 (65.9)	127 (61.1)	88 (71.5)	358 (65.3)
Employed	74 (34.1)	81 (38.9)	35 (28.5)	190 (34.7)
Woman education				
No education	109 (50.2)	2 (1.0)	0 (0.0)	111 (18.3)
Arabic education	25 (11.5)	0 (0.0)	0 (0.0)	25 (4.1)
Primary	24 (11.1)	10 (4.8)	8 (6.5)	42 (7.7)
Secondary	42 (19.4)	110 (52.9)	57 (46.3)	209 (38.1)
Tertiary	17 (7.8)	86 (41.4)	58 (47.2)	161 (29.4)
Husband's education				
No education	94 (43.3)	0 (0.0)	0 (0.0)	94 (17.2)
Primary	27 (12.4)	2 (1.0)	1 (0.8)	30 (5.5)
Secondary	43 (19.8)	89 (42.8)	48 (39.0)	100 (32.8)
Tertiary	53 (24.4)	117 (56.2)	74 (65.9)	244 (44.5)
Place of residence				
Rural	123 (56.7)	111 (53.4)	48 (39.0)	282 (51.5)
Urban	94 (43.3)	97 (46.6)	75 (71.0)	266 (48.5)
No of children ever born				
None/currently pregnant	10 (4.6)	78 (37.5)	44 (35.8)	132 (24.1)
1 or 2	72 (33.2)	95 (45.7)	58 (47.1)	225 (41.1)
3 and more	135 (62.2)	35 (16.8)	21 (17.1)	191 (34.8)
No of children alive				
None/currently pregnant	15 (6.9)	81 (38.9)	45 (36.6)	141 (25.7)
1 o 2	83 (38.3)	94 (45.2)	59 (48.0)	236 (43.1)
3 and more	119 (54.8)	33 (15.9)	19 (15.4)	171 (31.2)
Income*				
Undeclared	84 (38.7)	155 (74.5)	111 (90.2)	305 (63.9)
Less or equal to minimum wage	126 (58.1)	27 (13.0)	7 (5.7)	160 (29.2)
Greater than minimum wage	7 (3.2)	26 (12.5)	5 (4.1)	38 (6.9)
Wealth Index**				
Low	29 (13.4)	0 (0.0)	0 (0.0)	29 (5.3)
Middle	117 (53.9)	1 (0.5)	5 (4.1)	123 (22.5)
High	71 (32.7)	205 (99.5)	118 (95.9)	394 (72.2)

F, frequency.

* Minimum wage = 33,000 ($44 at $1 = 750).

** Missing wealth index = 2.

### Awareness and utilization of family planning methods

Overall, the proportion of women who reported to have used any family planning method before seeing a picture prompt was 43.8% (240/548), and this increased to 52.7% (289/548) after seeing the pictures. More women had used modern family planning methods (47.4%, 260/548) compared to traditional methods (18.2%, 100/548). There was regional variation in ever use of family planning, with 66.4% (138/208) of women in Lagos, 61.0% (75/123) in Oyo and only 35.0% (76/217) in Jigawa reporting use. Only 35.4% (194/548) of women reported to have used modern methods before their current pregnancy after seeing the pictures, which varied across the three states, with participants in Jigawa, Lagos and Oyo state 26.3% (57/217), 48.1% (100/208) and 30.1% (37/123) respectively.

Overall, 31.9% (175/548) respondents described their current pregnancy as unintended. Jigawa has the lowest proportion of unintended pregnancy at 22.1% (48/217), followed by Oyo state 28.5% (35/123), and Lagos 44.2% (92/208). In total, 363 (66.2%) were not using family planning methods before their current pregnancy and 26 (4.7%) reported their modern family planning method failed. Jigawa state has the highest proportion of 75.1% (163/217) women not using any family planning methods before their current pregnancy and lowest proportion of 27.7% (60/217) respondents willing to seek family planning advice after delivery. The primary sources of information about family planning were healthcare workers (83.8%), friends (35.8%), and family members (23.5%) (Table 2).

**Table 2:** Difference in awareness, utilization, motivation and failure of family planning methods among pregnant women between states.

Awareness and pattern of utilization of family planning method	Jigawa (*n* = 217)	Lagos (*n* = 208)	Oyo (*n* = 123)	Total (*N* = 548)
	F (%)	F (%)	F (%)	F (%)
Methods of FP Known without prompt*				
Traditional method—Yes	40 (18.4)	83 (39.9)	27 (22.0)	150 (27.4)
Modern method—Yes	212 (97.7)	190 (91.4)	116 (94.3)	518 (94.5)
Have you or your partner ever used any FP without prompt				
Yes	75 (34.6)	110 (52.9)	55 (44.7)	240 (43.8)
Ever used any family planning method to delay or space childbirth after seeing pictures				
Yes	76 (35.0)	138 (66.4)	75 (61.0)	289 (52.7)
Methods of FP ever used without prompt*				
Traditional method—Yes	2 (0.9)	27 (13.0)	12 (9.8)	41 (7.5)
Modern method—Yes	66 (30.4)	95 (45.7)	45 (36.6)	206 (37.6)
Methods of FP ever used after seeing picture*				
Traditional method—Yes	5 (2.3)	53 (25.5)	42 (34.1)	100 (18.3)
Modern method—Yes	75 (34.6)	127 (61.1)	58 (47.2)	260 (47.4)
Methods of FP recently used before current pregnancy after seeing picture*				
Traditional method—Yes	0 (0.0)	42 (20.2)	24 (19.5)	66 (12.0)
Modern method—Yes	57 (26.3)	100 (48.1)	37 (30.1)	194 (35.4)
Method of FP that failed (recent use) after picture*				
Traditional method—Yes	2 (0.9)	2 (1.0)	3 (2.4)	7 (1.3)
Modern method—Yes	11 (5.1)	12 (5.8)	3 (2.4)	26 (4.7)
Desire for the current pregnancy				
Respondent's desire to give birth -Yes	190 (87.6)	187 (89.9)	111 (90.2)	488 (89.1)
Partner's desire for the pregnancy—Yes	190 (87.6)	191 (91.8)	113 (91.9)	494 (90.2)
Prefer to have the pregnancy at a later time	48 (22.1)	92 (44.2)	35 (28.5)	175 (31.9)
Circumstances leading to current pregnancy°				
I stopped using family planning	39 (18.0)	73 (35.1)	38 (30.9)	150 (27.4)
I was not using any family planning method	163 (75.1)	121 (58.2)	79 (64.2)	363 (66.2)
The family planning method I was using failed	13 (6.0)	13 (6.3)	5 (4.1)	31 (5.7)
Willingness to go for family planning advice after your delivery	60 (27.7)	114 (54.8)	70 (56.9)	244 (44.5)
Method you would like to use after you deliver				
Traditional method—Yes	0 (0.0)	10 (4.8)	4 (3.3)	14 (2.6)
Modern method—Yes	46 (21.2)	74 (35.6)	29 (23.6)	149 (27.2)
Source of information about FP				
Partner	24 (11.1)	24 (11.5)	4 (3.3)	52 (9.5)
Friends	87 (40.1)	85 (40.9)	24 (19.5)	196 (35.8)
Family member	70 (32.3)	44 (21.2)	15 (12.2)	129 (23.5)
Neighbours	65 (30.0)	34 (16.4)	9 (7.3)	108 (19.7)
Radio and Television	34 (15.7)	59 (28.4)	8 (6.5)	101 (18.4)
Internet/social media	30 (13.8)	72 (34.6)	20 (16.3)	122 (22.3)
Health Care Worker	205 (94.5)	151 (72.6)	103 (83.7)	459 (83.8)
Posters/billboards or pamphlet	17 (7.8)	13 (6.3)	6 (4.9)	36 (6.6)
Informal healthcare providers	8 (3.7)	17 (8.2)	9 (7.3)	34 (6.2)

F, frequency.

* Multiple selection allowed.

° Missing circumstance prior current pregnancy (*n* = 4).

### Maternal health literacy

**Figure 1:**
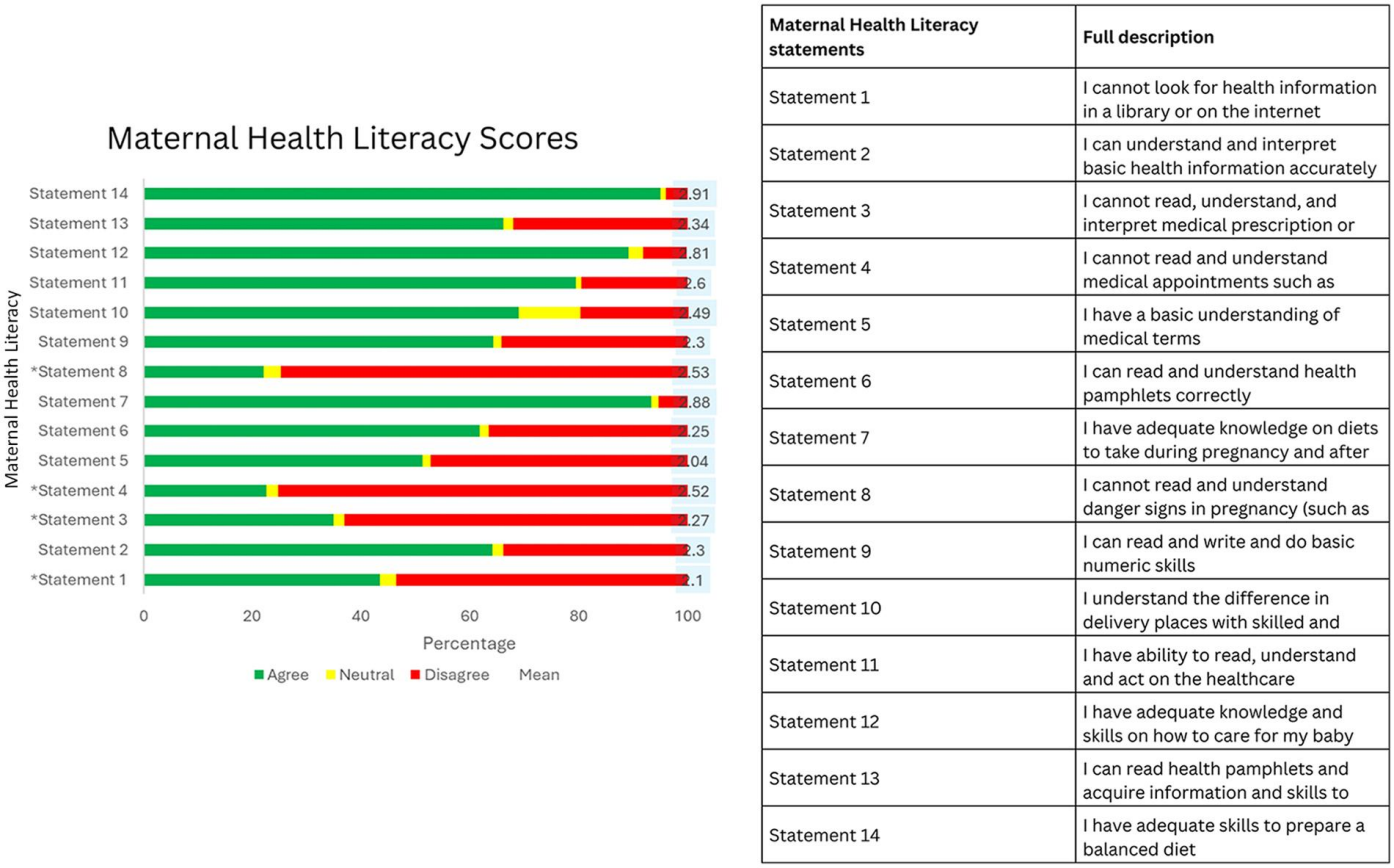
Maternal health literacy assessment results.

The mean maternal health literacy score across the study sample is 34.4 (SD ± 7.3) of a possible 42 (alpha coefficient = 0.8808). 133 respondents had a perfect score of 42 and only 2 respondents had the minimum score of 14. State-specific averages found Jigawa state had the lowest mean score of 30.1 (SD ± 6.4), with Lagos state had a mean score of 36.8 (SD ± 6.3), and Oyo state had a mean score 37.5 (SD ± 6.9). Most of the participants (95.6%) reported feeling confident in their ability to prepare a balanced diet, with an average score of 2.92 (SD ± 0.38) on this measure. Conversely, approximately half of the participants (51.3%) felt they had a basic understanding of medical terminology, indicated by a mean score of 2.04 (SD ± 0.99). State-wise, agreement with this statement varied: 24.0% in Jigawa, 64.9% in Lagos, and 74.2% in Oyo state. Additionally, a higher proportion of participants in Jigawa state (83.9%) disagreed with the statement, “I cannot read and understand danger signs in pregnancy (such as anemia, pallor, raised BP, swelling, bleeding, early labor, etc.)”, compared to 68.8% in Lagos and 69.1% in Oyo state (Figure 1 and Supplementary Appendix).

### Reasons why some women have never used any family planning methods

**Figure 2:**
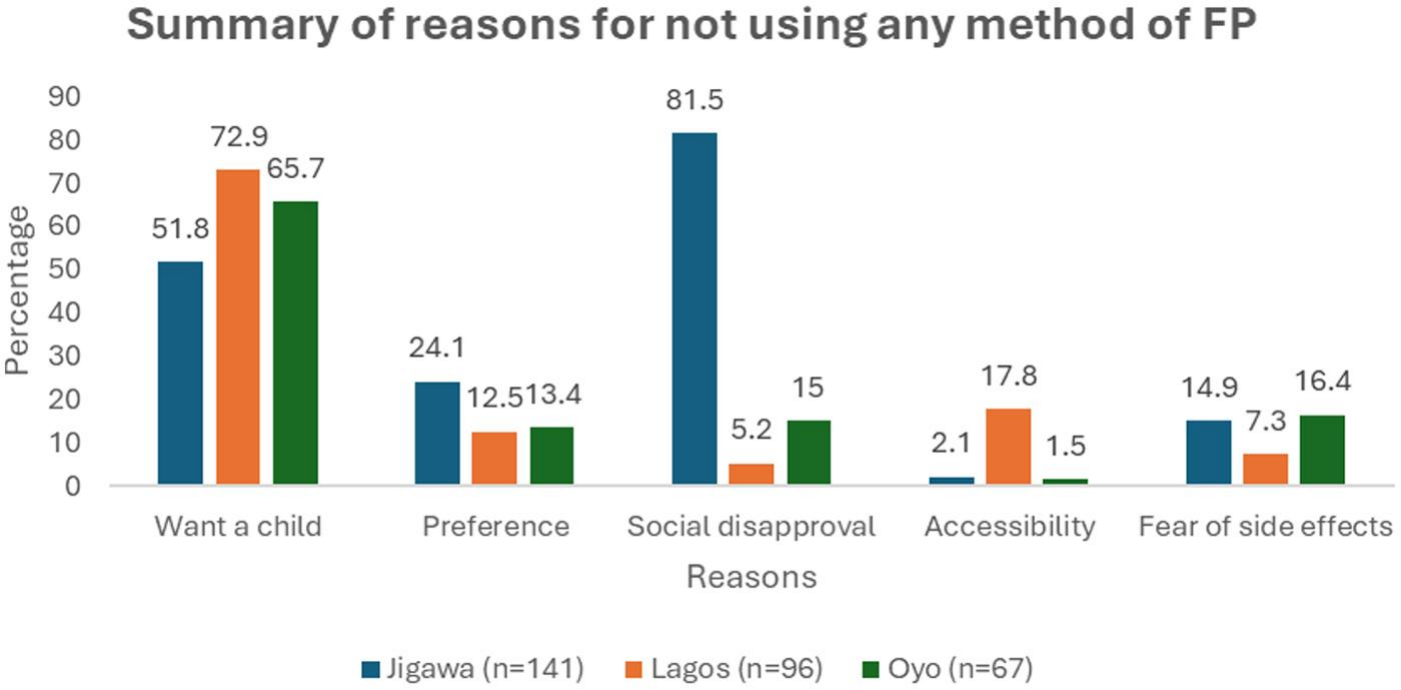
Reasons for non-use of any family planning methods.

The most common reason cited was the desire to have a child (Jigawa- 51.8%, Lagos- 72.9%, Oyo- 65.7%). Accessibility and fear of side effects were less frequently mentioned reasons. The distribution of these reasons varied slightly across the three states. In Jigawa, social disapproval (81.5%) significantly contributed to the lower uptake of family planning methods. In contrast, in Lagos, access barriers (17.8%) were a primary concern, while fears about side effects (7.3%) were less pronounced. Additionally, personal decision-making factors were more influential in Jigawa (24.1%), whereas a desire for children appeared to be more prevalent in the southern region (Figure 2).

### Association between use of family planning and MHL

The logistic regression analysis reveals significant associations between prior family planning use and women's MHL and other socio-demographic factors such as woman's age, woman's occupation, husband education, and husband occupation. Women who have at least a child alive have about twice the odds of having utilized family planning methods compared to women who don't have any child (1 child: aOR = 1.45, 95% CI: 0.78–2.66; 2–4 children: aOR = 2.93, 95% CI: 1.53–5.62; 5 and above children: aOR = 2.36, 95% CI: 0.80–6.94). Similarly, women from Lagos (aOR = 2.98, 95% CI: 1.27–7.01) and Oyo (aOR = 1.34, 95% CI: 0.52–3.44) states have about twice the odds of having utilized family planning methods compared to women in Jigawa state (Table 3).

**Table 3:** Logistic regression of ever use of any family planning method and maternal health literacy.

Variables		Unadjusted		Adjusted	
		OR	95% CI	OR	95% CI
Maternal Health Literacy		1.08	1.05–1.10	1.32	0.76–2.31
Age	15–24 years	Ref		Ref	
	25–34 years	1.73	1.12–2.67	1.09	0.62–1.92
	35–49 years	2.15	1.26–3.65	1.21	0.55–2.69
Religion	Christianity	Ref			
	Islam	0.34	0.23–0.49		
Ethnicity	Hausa- Fulani	Ref			
	Igbo	5.08	2.38–10.86		
	Yoruba	3.46	2.37–5.06		
	Others	11.40	3.78–34.39		
Woman's education	No formal education	Ref		Ref	
	Primary	2.04	0.98–4.22	1.52	0.57–4.09
	Secondary	4.55	2.82–7.34	1.19	0.43–3.25
	Tertiary	6.86	4.10–11.46	2.36	0.73–7.56
Husband's education	No formal education	Ref			
	Primary	2.28	0.93–5.60	2.45	0.93–6.47
	Secondary	3.94	2.21–7.06	1.49	0.55–4.01
	Tertiary	8.89	5.02–15.76	1.97	0.63–6.10
Woman occupation	Housewife/not working	Ref		Ref	
	Self employed	2.04	1.40–2.96	0.90	0.51–1.60
	Formal Employment	2.90	1.66–5.08	0.82	0.35–1.90
Husband occupation	Self employed	Ref		Ref	
	Formal Employment	2.12	1.48–3.05	1.26	0.71–2.22
Wealth Index	Low	Ref		Ref	
	Middle	1.76	0.62–4.99	2.43	0.81–7.31
	High	8.33	3.11–22.31	3.43	0.96–12.19
State	Jigawa	Ref		Ref	
	Lagos	3.65	2.45–5.46	2.98	1.27–7.01
	Oyo	2.90	1.83–4.58	1.34	0.52–3.44
Children alive	None	Ref		Ref	
	Only one child	1.21	0.73–2.01	1.45	0.78–2.66
	2–4 children	1.64	1.04–2.61	2.95	1.53–5.62
	5 and above children	0.55	0.29–1.03	2.36	0.80–6.94

## Discussion

We assessed family planning utilisation, barriers to access, and motivations among pregnant women in Nigeria, with a focus on the association with maternal health literacy (MHL). The main reason cited for not using family planning methods was a desire to become pregnant, as many women knew about family planning options but did not use them before their current pregnancy. Approximately half of the women had used a modern family planning method, and about one-quarter achieved a perfect MHL score, with healthcare workers being the primary source of family planning information. However, involvement from husbands in family planning discussions was limited. Additionally, the use of family planning methods was more common among women who had at least one living child and those from tribes other than Hausa/Fulani. We noticed some regional variation, as desire for more children was notably higher in the south: Lagos and Oyo states, contributing to lower family planning use. In Jigawa, social disapproval and personal decision-making were significant barriers, while in Lagos, access issues were prominent; however, fear of side effects was less prevalent, suggesting better knowledge and acceptance.

Our findings align with data from the National Demographic and Health Survey (NDHS), which indicates that contraceptive prevalence in Nigeria remains relatively low. We found a significant association between a knowledge of family planning methods and prior usage, consistent with findings from other studies (13, 41). Nonetheless, substantial barriers persist that hinder access and utilization of family planning services, including cultural beliefs, stigma, lack of formal education, and religious influences, particularly in Jigawa, which contribute to a reluctance to seek such services (42, 43). This resulted in an observed gap between awareness and actual use of family planning methods, with variations in usage between traditional and modern methods (13, 44). We recommend implementing targeted behavioral interventions in Jigawa to address these challenges.

Our results indicated a notable relationship between maternal health literacy (MHL) and family planning uptake in the unadjusted model; each one-point increase in MHL was associated with an 8% higher likelihood of using family planning methods. However, this association was influenced by other sociodemographic factors, such as maternal education, when adjustments were made in the model. Research indicates that elevated levels of maternal health literacy (MHL) are strongly correlated with better utilization of maternal healthcare services and effective family planning practices, including the adoption of postpartum contraceptives (39, 45). Similarly, Yaya et al. emphasize that maternal health literacy significantly empowers women by improving their ability to access and comprehend available family planning options, ultimately leading to improved uptake rates (46). Furthermore, high levels of MHL are associated with favorable pregnancy outcomes, which include increased neonatal birth weight, enhanced maternal health indicators, and higher rates of breastfeeding (47, 48). Concurrently, the engagement with healthcare facility services, such as antenatal care, reinforces the role of healthcare providers as essential sources of family planning information. This dynamic has been positively associated with heightened awareness among pregnant women regarding diverse family planning methods (49).

Regional variations in family planning uptake in Nigeria highlight the need for context-specific interventions. A common reason cited by women for not using or discontinuing family planning is the desire to have more children, which seems higher in the south. This may be due to the rising incidence of infertility in the south compared to the north, which has been linked to a decline in contraceptive use, as individuals may perceive a lesser need for contraception when facing difficulties with conception (50–54). It could also be because the inclination to have more children is often rooted in cultural and religious norms that favour larger family sizes, which are often associated with prestige and social status, particularly in polygynous communities (13, 55, 56). Moreover, the belief that having multiple children serves as a safeguard against child mortality, especially in regions with high child mortality rates like Jigawa State, reinforces this perspective (57, 58). For some women, the strong desire to become mothers or expand their families, particularly in the absence of full-time employment, is tied to personal identity and a sense of fulfillment of motherhood. This motivation helps explain why desire for pregnancy is more commonly mentioned as a reason for non-use in Oyo and Lagos than in Jigawa, as about 36% of women in Oyo and Lagos are pregnant with their first child compared with about 5% in Jigawa (59–61). In addition, high under-five mortality rate, low life expectancy, and lack of social protection or support in Nigeria may influence many couples to desire to have more than one child (62, 63). This means that improvement in family planning uptake requires more than accessibility and health education. Other broader systemic issues require improvement for sustained and improved family planning uptake. Additionally, sociocultural preferences appear to influence reproductive decisions; even couples satisfied with their current family size may passively accept the potential for having another child, especially when they have not yet welcomed a male child (64, 65). These regional variations may be reflective of actual demographic shifts or influence of cultural factors in shaping contraceptive behavior, which highlight the need for tailored public health strategies and educational initiatives for a better reproductive health outcome.

In Jigawa, social disapproval emerged as a significant barrier to contraceptive use, reflecting broader cultural attitudes that may stigmatize family planning practices as identified in other Sub-Saharan Africa contexts (66, 67). Social norms that discourage contraception negatively impact its use by primarily affecting women's ability to access and use contraceptives irrespective of their intent or motivation (68). Disapproval from a spouse or relative might stem from deeper cultural, religious, or personal beliefs, potentially leading to conflicts and pressures within relationships. Conversely, increased contraceptive use has been associated with residing in ethnically diverse communities and areas with higher levels of education on average (69, 70). Interventions that modify norms for a better reproductive health outcome is suggested.

Furthermore, our findings indicate that individual preferences and social norms hostile to family planning play a crucial role in the utilization of family planning methods in Jigawa. This observation is consistent with broader research suggesting that women's active participation in household decision-making processes is positively correlated with increased use of modern contraceptives. Studies have shown that when women are empowered to make autonomous decisions regarding their reproductive health, they are more likely to adopt and consistently use effective family planning methods (71). Such empowerment can lead to more informed choices, better access to contraceptive services, and a greater ability to manage reproductive health. Thus, enhancing women's roles in decision-making through targeted behavioral change interventions, not only supports their reproductive autonomy but also contributes to improved family planning outcomes, maternal health and reduced child mortality (72–74). The observed suboptimal use of family planning methods, despite high health literacy among pregnant women, points to potential limitations in women's empowerment, which may impede progress toward achieving Sustainable Development Goal 5 (SDG-5) on gender equality and women's empowerment (75). This implies that decisions about family planning may be influenced by variables other than knowledge alone. While healthcare workers have been identified as the primary source of family planning information, partners were ranked among the least utilized sources, highlighting a concerning lack of spousal communication regarding family planning (76, 77). Anecdotal evidence suggest that cultural norms often place the burden of initiating family planning discussions on husbands, leading to perceptions that a woman's initiative in such discussions could be seen as disloyal or indicative of a lack of commitment to her marriage (78). These cultural barriers may deter women from initiating family planning conversations or using contraceptive methods. To address these issues effectively, future family planning interventions should focus on enhancing male involvement and engagement in discussions. By fostering open communication between partners and promoting shared decision-making, we can better empower women and improve family planning outcomes.

In contrast, Lagos encounters significant challenges related to access to family planning services, primarily due to its high population density and the logistical issues that accompany it. The state's growing population results in a demand for family planning services that often exceeds the available supply, exacerbating issues related to accessibility and service delivery. Additionally, the disparities in contraceptive use in Lagos are influenced by socioeconomic factors, as it has more higher-income women, showing a greater likelihood of using contraceptives compared to other states (79). Despite these challenges, Lagos reports lower levels of fear regarding side effects, which may be attributed to higher levels of knowledge and acceptance of family planning methods. Overall, these regional disparities emphasize that interventions must be tailored to address specific local barriers and contexts, ensuring that family planning programs are both relevant and effective in diverse settings across Nigeria.

## Strengths and limitations

The study has some few limitations. First, we selected currently pregnant women attending health facilities. There is a risk of selection bias as views from other pregnant women in the communities were not captured. There is also possibility of recall bias and the team was not able to ascertain family utilization uptake. Despite these limitations, the use of pictures and data collection across states in different geopolitical zones helps to capture nuanced differences that are relevant to developing interventions to improve family planning uptake.

## Conclusion

Family planning utilization differs across settings in Nigeria, and it is shaped by contextual factors such as social and cultural norms as well as fertility rates. Improvement in family planning uptake requires more than just availability. Women's empowerment and a broader system and human capital development are necessary to improve utilization. These findings highlight the need for context-specific family planning interventions in Nigeria, including strengthening antenatal counselling, addressing socio-cultural barriers, and improving women's empowerment to enhance family planning uptake.

## Data Availability

The raw data supporting the conclusions of this article will be made available by the authors, without undue reservation.
