# Supplementary material for: Assessing patterns, barriers, and motivations for family planning utilization among currently pregnant women in Nigeria: a cross-sectional study
Source: Front Reprod Health. 2026 May 21;8:1789800. doi: 10.3389/frph.2026.1789800 (PMC13233478; doi:10.3389/frph.2026.1789800)
Supplement: Supplementary file 1 [file Table1.docx]

**Supplementary material 2: Maternal health literacy of pregnant women (N=548)**

| **Maternal health literacy** | **Agree** | **Neutral** | **Disagree** | **Mean** | **±SD** |
| --- | --- | --- | --- | --- | --- |
| I cannot look for health information in a library or on the internet | 238 (43.4) | 17 (3.1) | 293 (53.5) | 2.10 | 0.98 |
| I can understand and interpret basic health information accurately | 351 (64.1) | 11 (2.0) | 186 (33.9) | 2.30 | 0.94 |
| I cannot read, understand, and interpret medical prescription or instructions accurately | 192 (35.0) | 11 (2.0) | 345 (63.0) | 2.27 | 0.95 |
| I cannot read and understand medical appointments such as dates for immunization screening, physical examination | 124 (22.6) | 12 (2.2) | 412 (75.2) | 2.52 | 0.84 |
| I have a basic understanding of medical terms | 281 (51.3) | 9 (1.6) | 258 (47.1) | 2.04 | 0.99 |
| I can read and understand health pamphlets correctly | 339 (61.9) | 9 (1.6) | 200 (36.5) | 2.25 | 0.96 |
| I have adequate knowledge on diets to take during pregnancy and after delivery | 512 (93.4) | 7 (1.3) | 29 (5.3) | 2.88 | 0.46 |
| I cannot read and understand danger signs in pregnancy (such as Anaemia, pallor, raised BP, swelling, bleeding, early labour e.t.c | 121 (22.1) | 17 (3.1) | 410 (74.8) | 2.53 | 0.83 |
| I can read and write and do basic numeric skills | 353 (64.4) | 8 (1.5) | 187 (34.1) | 2.30 | 0.95 |
| I understand the difference in delivery places with skilled and those without skilled birth attendants | 378 (69.0) | 61 (11.3) | 109 (19.9) | 2.49 | 0.81 |
| I have ability to read, understand and act on the healthcare information positively | 436 (79.6) | 5 (0.9) | 107 (19.5) | 2.60 | 0.79 |
| I have adequate knowledge and skills on how to care for my baby after delivery (breastfeeding, bathing) | 489 (89.2) | 15 (2.7) | 44 (8.0) | 2.81 | 0.56 |
| I can read health pamphlets and acquire information and skills to maintain personal and food hygiene during pregnancy and after delivery | 362 (66.1) | 11 (2.0) | 175 (31.9) | 2.34 | 0.93 |
| I have adequate skills to prepare a balanced diet | 521 (95.1) | 5 (0.9) | 22 (4.0) | 2.91 | 0.40 |
