# Supplementary material for: Assessing patterns, barriers, and motivations for family planning utilization among currently pregnant women in Nigeria: a cross-sectional study
Source: Front Reprod Health. 2026 May 21;8:1789800. doi: 10.3389/frph.2026.1789800 (PMC13233478; doi:10.3389/frph.2026.1789800)
Supplement: Supplementary file 2 [file Table2.docx]

**Supplementary material 3: Reasons for non-use of any family planning methods**

| **Reasons for non-use** |  | **Jigawa**  **(n=141)** | **Lagos**  **(n=96)** | **Oyo**  **(n=67)** | **Total**  **(n=304)** | **Chi-square p-value** |
| --- | --- | --- | --- | --- | --- | --- |
| **Do not need it/want a child (C)** | Yes | 73 (51.8) | 70 (72.9) | 44 (65.7) | 187 (61.5) | 0.003 |
|  | No | 68 (48.2) | 26 (27.1) | 23 (34.3) | 117 (38.5) |  |
| **I do not like it (P)** | Yes | 32 (22.7) | 10 (10.4) | 9 (13.4) | 51 (16.8) | 0.032 |
|  | No | 109 (77.3) | 86 (89.6) | 58 (86.6) | 253 (83.2) |  |
| **It is against my religious belief (S)** | Yes | 4 (2.8) | 2 (2.1) | 3 (4.5) | 9 (3.0) | 0.670 |
|  | No | 137 (97.2) | 94 (97.9) | 64 (95.5) | 295 (97.0) |  |
| **My partner is not in support (S)** | Yes | 85 (60.3) | 3 (3.1) | 5 (7.5) | 93 (30.6) | <0.001 |
|  | No | 56 (39.7) | 93 (96.9) | 62 (92.5) | 211 (69.4) |  |
| **My partner’s family are not in support (S)** | Yes | 26 (18.4) | 0 (0.0) | 2 (3.0) | 28 (9.2) | <0.001 |
|  | No | 115 (81.6) | 96 (100.0) | 65 (97.0) | 276 (90.8) |  |
| **It does not work for me(P)** | Yes | 2 (1.4) | 2 (2.1) | 0 (0.0) | 4 (1.3) | 0.512 |
|  | No | 139 (98.6) | 94 (97.9) | 67 (100.0) | 300 (98.7) |  |
| **It is expensive (A)** | Yes | 0 (0.0) | 0 (0.0) | 1 (1.5) | 1 (0.3) | 0.170 |
|  | No | 141 (100.0) | 96 (100.0) | 66 (98.5) | 303 (99.7) |  |
| **It is not accessible to me (A)** | Yes | 0 (0.0) | 9 (9.4) | 0 (0.0) | 9 (3.0) | <0.001 |
|  | No | 141 (100.0) | 87 (90.6) | 67 (100.0) | 295 (97.0) |  |
| **My parents did not use it (F)** | Yes | 3 (2.1) | 0 (0.0) | 1 (1.5) | 4 (1.3) | 0.366 |
|  | No | 138 (97.9) | 96 (100.0) | 66 (98.5) | 300 (98.7) |  |
| **I am afraid of the side effects (F)** | Yes | 18 (12.8) | 7 (7.3) | 10 (14.9) | 35 (11.5) | 0.264 |
|  | No | 123 (87.2) | 89 (92.7) | 57 (85.1) | 269 (88.5) |  |
| **Not accessible to my partner (A)** | Yes | 0 (0.0) | 4 (4.2) | 0 (0.0) | 4 (1.3) | 0.012 |
|  | No | 141 (100.0) | 92 (95.8) | 67 (100.0) | 300 (98.7) |  |
| **Nobody to train me or my partner (A)** | Yes | 3 (2.1) | 4 (4.2) | 0 (0.0) | 7 (2.3) | 0.214 |
|  | No | 138 (97.9) | 92 (95.8) | 67 (100.0) | 297 (97.7) |  |
| **Others** | Yes | 3 (2.1) | 2 (2.1) | 10 (14.9) | 15 (4.9) | <0.001 |
|  | No | 138 (97.9) | 94 (97.9) | 57 (85.1) | 289 (95.1) |  |

**C=Child P= Preference S=Social disapproval A=Accessibility F=Fear of side effect**
