# Supplementary material for: Assessing patterns, barriers, and motivations for family planning utilization among currently pregnant women in Nigeria: a cross-sectional study
Source: Front Reprod Health. 2026 May 21;8:1789800. doi: 10.3389/frph.2026.1789800 (PMC13233478; doi:10.3389/frph.2026.1789800)
Supplement: Supplementary file 3 [file Table3.docx]

**Supplementary material 4: Logistic regression of ever use of any family planning method and MHL while clustering for facilities in Jigawa**

| **Multiple logistic regression** | | **Unadjusted** | | | | **Adjusted** | | | |
| --- | --- | --- | --- | --- | --- | --- | --- | --- | --- |
| **Variables** | | **odds ratio** | **95% confidence interval** | | **p-value** | **odds ratio** | **95% confidence interval** | | **p-value** |
| MHL |  | 1.10 | (1.02 | 1.17) | 0.008 | 0.91 | (0.83 | 0.99) | 0.038 |
| Age | 15-24 years | Ref |  |  |  | Ref |  |  |  |
|  | 25-34 years | 1.87 | (0.91 | 3.87) | 0.090 | 1.31 | (0.66 | 2.60) | 0.431 |
|  | 35-49 years | 1.28 | (0.53 | 3.08) | 0.587 | 1.36 | (0.36 | 5.17) | 0.644 |
| Religion | Christianity | Ref |  |  |  | Ref |  |  |  |
|  | Islam | 0.08 | (0.02 | 0.41) | 0.002 | 0.19 | (0.05 | 0.61) | 0.005 |
| Woman’s education | No formal education | Ref |  |  |  | Ref |  |  |  |
|  | Primary | 0.81 | (0.41 | 1.59) | 0.536 | 0.70 | (0.34 | 1.41) | 0.321 |
|  | Secondary | 4.97 | (2.01 | 12.29) | 0.001 | 11.27 | (1.90 | 66.87) | 0.008 |
|  | Tertiary | 7.34 | (2.08 | 25.87) | 0.002 | 17.72 | (2.70 | 166.12) | 0.003 |
| Husband’s education | No formal education | Ref |  |  |  |  |  |  |  |
|  | Primary | 1.97 | (1.22 | 3.18) | 0.005 | 2.88 | (1.53 | 5.39) | 0.001 |
|  | Secondary | 2.58 | (1.14 | 5.82) | 0.022 | 2.21 | (0.64 | 7.69) | 0.211 |
|  | Tertiary | 5.56 | (1.95 | 15.83) | 0.001 | 1.52 | (0.12 | 19.40) | 0.747 |
| Woman occupation | Housewife/notworking | Ref |  |  |  | Ref |  |  |  |
|  | Self employed | 0.88 | (0.43 | 1.81) | 0.730 | 0.64 | (0.27 | 1.49) | 0.303 |
|  | Formal Employment | 4.45 | (0.99 | 19.95) | 0.051 | 0.56 | (0.14 | 2.22) | 0.414 |
| Husband occupation | Self employed | Ref |  |  |  | Ref |  |  |  |
|  | Formal Employment | 2.67 | (1.21 | 5.89) | 0.015 | 0.78 | (0.27 | 2.22) | 0.635 |
| Wealth Index | Low | Ref |  |  |  | Ref |  |  |  |
|  | Middle | 1.65 | (0.53 | 5.17) | 0.387 | 1.12 | (0.30 | 4.16) | 0.856 |
|  | High | 6.56 | (2.80 | 15.34) | <0.001 | 2.26 | (0.57 | 8.88) | **0.239** |
| Children alive | None | Ref |  |  |  | Ref |  |  |  |
|  | Only one child | 3.56 | (1.02 | 12.50) | 0.047 | 7.22 | (1.43 | 36.49) | **0.017** |
|  | 2-4 children | 4.61 | (1.45 | 14.60) | 0.009 | 12.61 | (2.21 | 71.64) | **0.004** |
|  | 5 and above children | 2.78 | (0.73 | 10.67) | 0.135 | 9.10 | (1.46 | 56.58) | **0.018** |
