# Supplementary material for: Assessing patterns, barriers, and motivations for family planning utilization among currently pregnant women in Nigeria: a cross-sectional study
Source: Front Reprod Health. 2026 May 21;8:1789800. doi: 10.3389/frph.2026.1789800 (PMC13233478; doi:10.3389/frph.2026.1789800)
Supplement: Supplementary file 4 [file Table4.docx]

**Supplementary material 5: Logistic regression of ever use of any family planning method and MHL while clustering for facilities in Lagos**

| **Multiple logistic regression** | | **Unadjusted** | | | | **Adjusted** | | | |
| --- | --- | --- | --- | --- | --- | --- | --- | --- | --- |
| **Variables** | | **odds ratio** | **95% confidence interval** | | **p-value** | **odds ratio** | **95% confidence interval** | | **p-value** |
| MHL |  | 1.01 | (0.93 | 1.10) | 0.837 | 0.99 | (0.92 | 1.08) | 0.999 |
| Age | 15-24 years | Ref |  |  |  | Ref |  |  |  |
|  | 25-34 years | 0.83 | (0.19 | 3.64) | 0.805 | 0.72 | (0.15 | 3.44) | 0.683 |
|  | 35-49 years | 1.44 | (0.38 | 5.42) | 0.590 | 1.23 | (0.32 | 4.66) | 0.751 |
| Religion | Christianity | Ref |  |  |  | Ref |  |  |  |
|  | Islam | 0.59 | (0.42 | 0.83) | 0.002 | 0.64 | (0.42 | 0.95) | 0.031 |
| Woman’s education | No formal education | Ref |  |  |  | Ref |  |  |  |
|  | Primary | 3.99 | (0.33 | 47.06) | 0.270 | 3.85 | (0.27 | 53.78) | 0.317 |
|  | Secondary | 1.68 | (0.13 | 21.21) | 0.687 | 1.58 | (0.12 | 19.14) | 0.721 |
|  | Tertiary | 2.31 | (0.13 | 40.20) | 0.566 | 1.78 | (0.16 | 19.25) | 0.633 |
| Woman occupation | Housewife/notworking | Ref |  |  |  | Ref |  |  |  |
|  | Self employed | 1.08 | (0.46 | 2.51) | 0.856 | 1.11 | (0.47 | 2.59) | 0.815 |
|  | Formal Employment | 1.39 | (0.44 | 4.35) | 0.574 | 1.02 | (0.46 | 2.26) | 0.954 |
| Husband occupation | Self employed | Ref |  |  |  | Ref |  |  |  |
|  | Formal Employment | 1.79 | (0.90 | 3.57) | 0.096 | 1.80 | (0.81 | 4.02) | 0.151 |
