# Supplementary material for: Assessing patterns, barriers, and motivations for family planning utilization among currently pregnant women in Nigeria: a cross-sectional study
Source: Front Reprod Health. 2026 May 21;8:1789800. doi: 10.3389/frph.2026.1789800 (PMC13233478; doi:10.3389/frph.2026.1789800)
Supplement: Supplementary file 5 [file Table5.docx]

**Supplementary material 6: Logistic regression of ever use of any family planning method and MHL while clustering for facilities in Oyo**

| **Multiple logistic regression** | | **Unadjusted** | | | | **Adjusted** | | | |
| --- | --- | --- | --- | --- | --- | --- | --- | --- | --- |
| **Variables** | | **odds ratio** | **95% confidence interval** | | **p-value** | **odds ratio** | **95% confidence interval** | | **p-value** |
| MHL |  | 1.04 | (0.99 | 1.09) | 0.151 | 1.03 | (0.95 | 1.13) | 0.401 |
| Age | 15-24 years | Ref |  |  |  | Ref |  |  |  |
|  | 25-34 years | 2.81 | (1.66 | 4.76) | <0.001 | 2.56 | (1.41 | 4.68) | 0.002 |
|  | 35-49 years | 6.02 | (2.12 | 17.08) | 0.001 | 6.35 | (1.94 | 20.75) | 0.002 |
| Religion | Christianity | Ref |  |  |  | Ref |  |  |  |
|  | Islam | 0.87 | (0.48 | 1.58) | 0.652 | 1.30 | (0.71 | 2.38) | 0.382 |
| Ethnicity | Hausa- Fulani | Ref |  |  |  | Ref |  |  |  |
|  | Igbo | 1.00 | (0.02 | 52.49) | 1.000 | 0.66 | (0.01 | 249.11) | 0.891 |
|  | Yoruba | 1.55 | (0.05 | 48.14) | 0.803 | 1.45 | (0.01 | 281.92) | 0.889 |
|  | Others | 4.00 | (0.26 | 59.43) | 0.314 | 3.25 | (0.02 | 427.05) | 0.636 |
| Woman’s education | Primary | Ref |  |  |  | Ref |  |  |  |
|  | Secondary | 0.93 | (0.22 | 3.95) | 0.922 | 1.00 | (0.18 | 5.59) | 0.998 |
|  | Tertiary | 1.54 | (0.36 | 6.53) | 0.557 | 1.43 | (0.12 | 16.23) | 0.768 |
| Woman occupation | Housewife/notworking | Ref |  |  |  | Ref |  |  |  |
|  | Self employed | 0.94 | (0.39 | 2.27) | 0.896 | 1.31 | (0.44 | 3.93) | 0.619 |
|  | Formal Employment | 0.90 | (0.44 | 1.82) | 0.770 | 0.78 | (0.32 | 1.83) | 0.564 |
| Husband occupation | Self employed | Ref |  |  |  | Ref |  |  |  |
|  | Formal Employment | 2.05 | (1.34 | 3.14) | 0.001 | 2.40 | (1.31 | 4.43) | 0.005 |
| Wealth Index | Middle | Ref |  |  |  | Ref |  |  |  |
|  | High | 1.04 | (0.17 | 6.52) | 0.962 | 0.57 | (0.123 | 2.69) | 0.485 |
