# Supplementary material for: Assessing patterns, barriers, and motivations for family planning utilization among currently pregnant women in Nigeria: a cross-sectional study
Source: Front Reprod Health. 2026 May 21;8:1789800. doi: 10.3389/frph.2026.1789800 (PMC13233478; doi:10.3389/frph.2026.1789800)
Supplement: Supplementary file 6 [file Table6.docx]

| **Source of information about FP** | **Jigawa (n=217)** | **Lagos (n=208)** | **Oyo (n=123)** | **Total**  **(N= 548)** |
| --- | --- | --- | --- | --- |
| Partner | 24 (11.1) | 24 (11.5) | 4 (3.3) | 52 (9.5) |
| Friends | 87 (40.1) | 85 (40.9) | 24 (19.5) | 196 (35.8) |
| Family member | 70 (32.3) | 44 (21.2) | 15 (12.2) | 129 (23.5) |
| Neighbours | 65 (30.0) | 34 (16.4) | 9 (7.3) | 108 (19.7) |
| Radio and Television | 34 (15.7) | 59 (28.4) | 8 (6.5) | 101 (18.4) |
| Internet/social media | 30 (13.8) | 72 (34.6) | 20 (16.3) | 122 (22.3) |
| Health Care Worker | 205 (94.5) | 151 (72.6) | 103 (83.7) | 459 (83.8) |
| Posters/billboards or pamphlet | 17 (7.8) | 13 (6.3) | 6 (4.9) | 36 (6.6) |
| Informal healthcare providers | 8 (3.7) | 17 (8.2) | 9 (7.3) | 34 (6.2) |

**Appendix**
