# Supplementary material for: Assessing patterns, barriers, and motivations for family planning utilization among currently pregnant women in Nigeria: a cross-sectional study
Source: Front Reprod Health. 2026 May 21;8:1789800. doi: 10.3389/frph.2026.1789800 (PMC13233478; doi:10.3389/frph.2026.1789800)
Supplement: Supplementary file 7 [file Datasheet1.pdf]

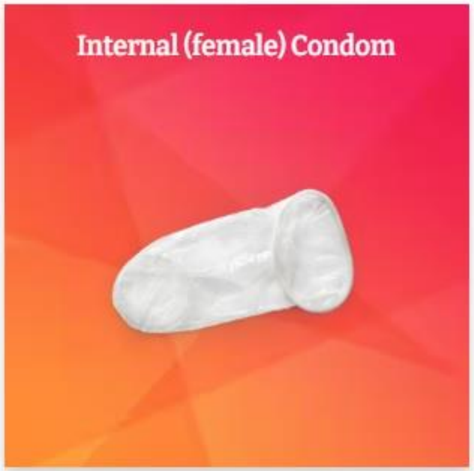

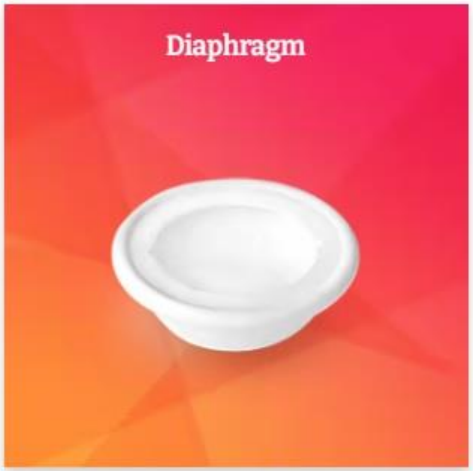

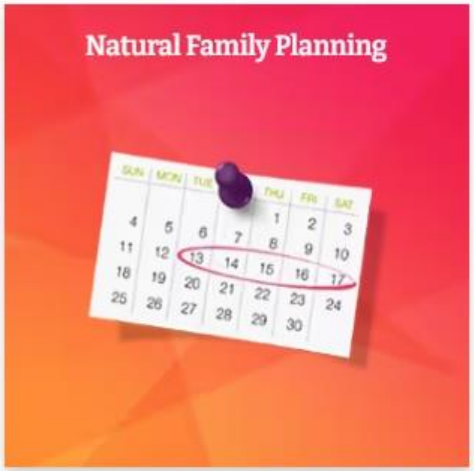

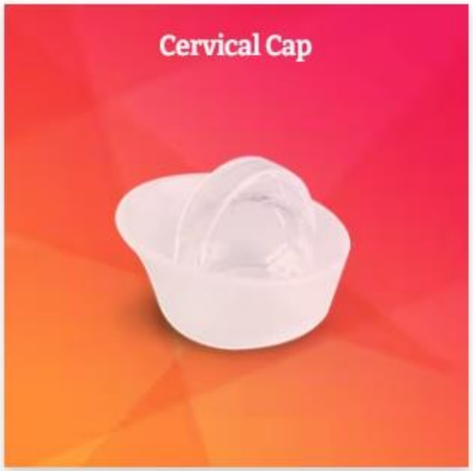

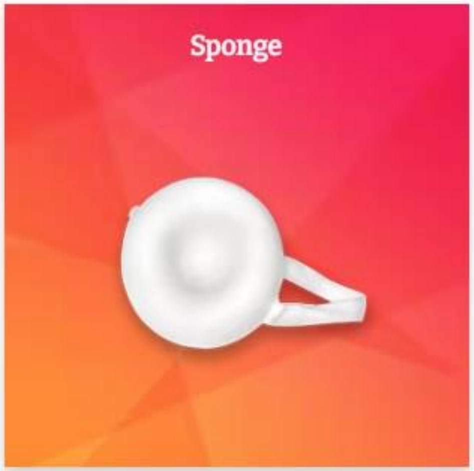

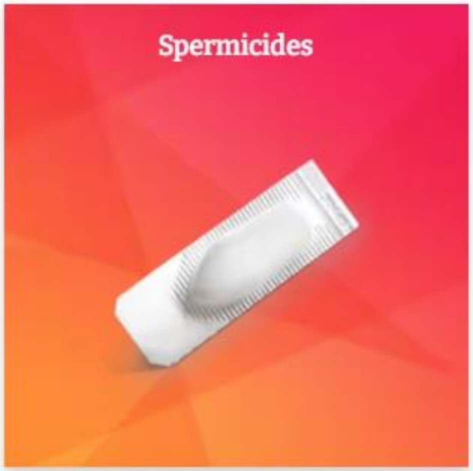

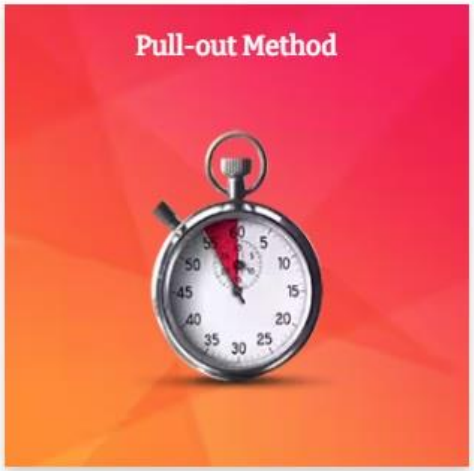

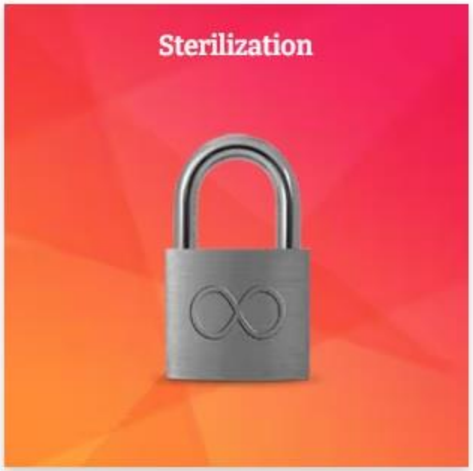

Source: https://www.nhs.uk/conditions/contraception/contraceptive-injection/; https://www.your-life.com/en/contraception-methods; https://unsplash.com/s/photos/contraception

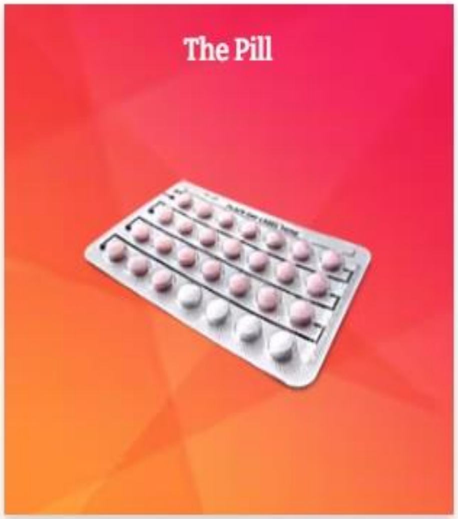

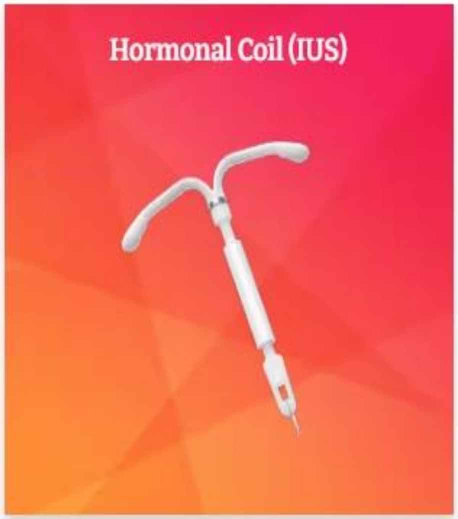

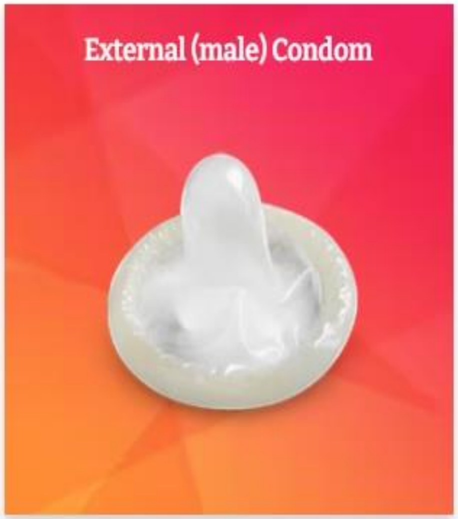

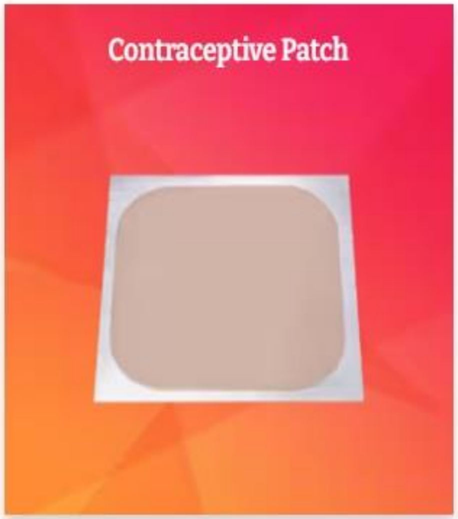

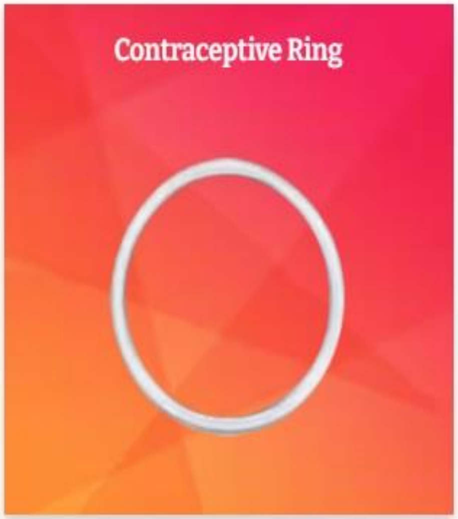

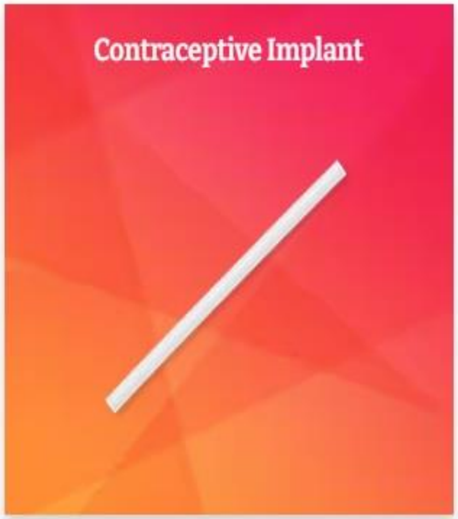

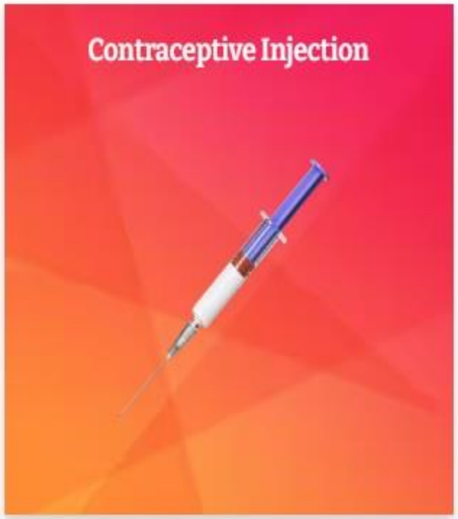

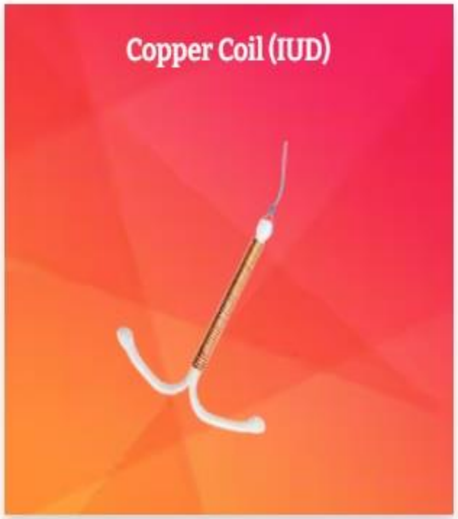

[illegible], [illegible],
https://unsplash.com/s/photos/contraception

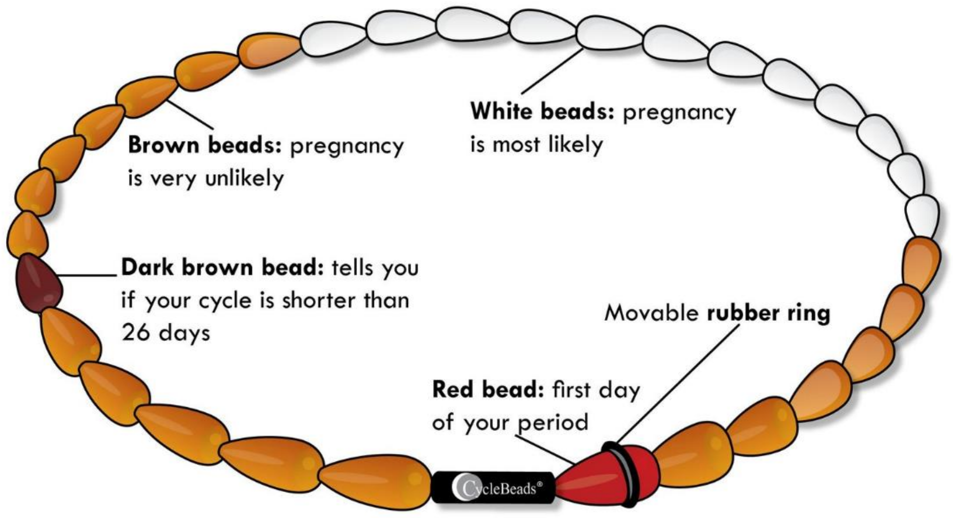

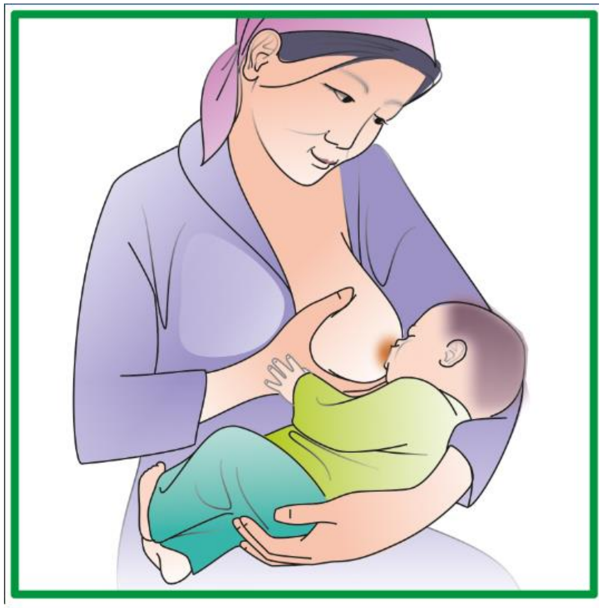

WITHDRAWAL METHODS

Source: https://www.nhs.uk/conditions/contraception/contraceptive-injection/; https://www.your-life.com/en/contraception-methods; https://unsplash.com/s/photos/contraception

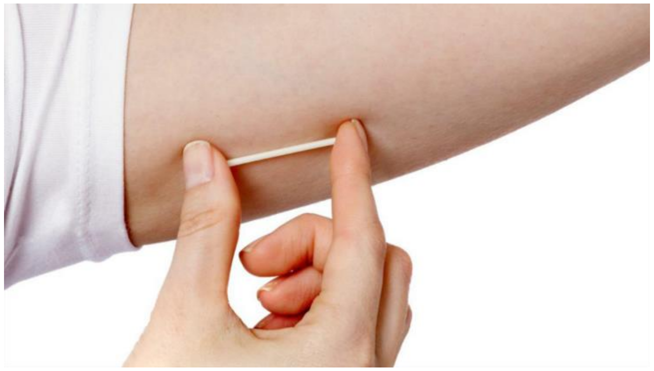

IMPLANT

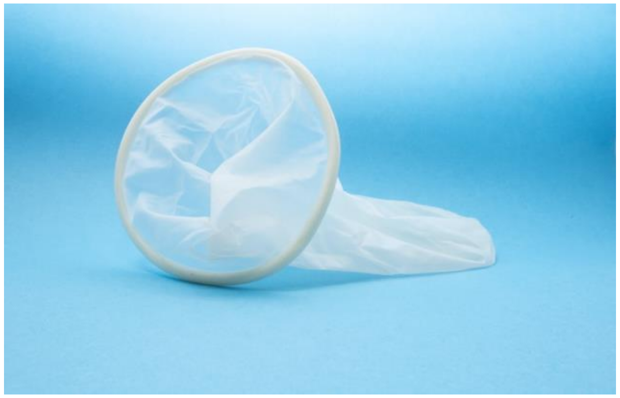

MALE CONDOM

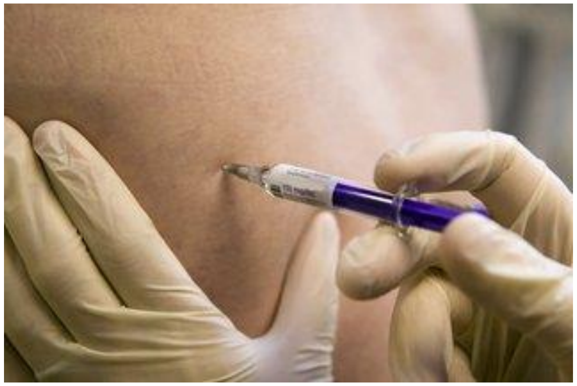

INJECTION

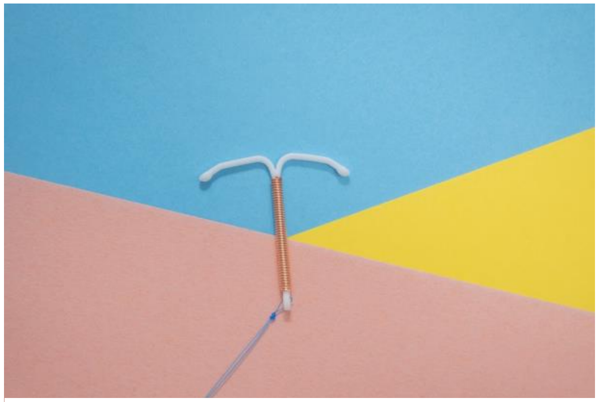

INTRA-UTERINE DEVICE

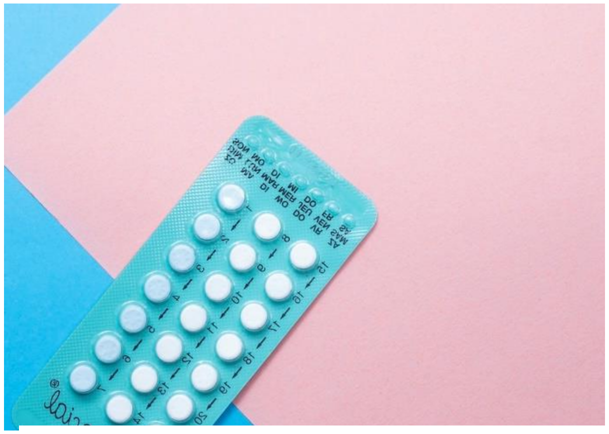

ORAL CONTRACEPTIVE PILLS

Source: https://www.nhs.uk/conditions/contraception/contraceptive-injection/; https://www.your-life.com/en/contraception-methods; https://unsplash.com/s/photos/contraception
